# Carnitine Inborn Errors of Metabolism

**DOI:** 10.3390/molecules24183251

**Published:** 2019-09-06

**Authors:** Mohammed Almannai, Majid Alfadhel, Ayman W. El-Hattab

**Affiliations:** 1 Section of Medical Genetics, Children’s Hospital, King Fahad Medical City, Riyadh 11525, Saudi Arabia; 2 Division of Genetics, Department of Pediatrics, King Abdulaziz Medical City, Ministry of National Guard-Health Affairs (MNGHA), Riyadh 11426, Saudi Arabia; 3 King Abdullah International Medical Research Center (KAIMRC), Riyadh 11426, Saudi Arabia; 4 College of Medicine, King Saud Bin Abdulaziz University for Health Sciences, Riyadh 11426, Saudi Arabia; 5 Department of Clinical Sciences, College of Medicine, University of Sharjah, Sharjah 27272, UAE

**Keywords:** carnitine, trimethyllysine (TML) dioxygenase, carnitine transporter, carnitine palmitoyltransferase

## Abstract

Carnitine plays essential roles in intermediary metabolism. In non-vegetarians, most of carnitine sources (~75%) are obtained from diet whereas endogenous synthesis accounts for around 25%. Renal carnitine reabsorption along with dietary intake and endogenous production maintain carnitine homeostasis. The precursors for carnitine biosynthesis are lysine and methionine. The biosynthetic pathway involves four enzymes: 6-*N*-trimethyllysine dioxygenase (TMLD), 3-hydroxy-6-*N*-trimethyllysine aldolase (HTMLA), 4-*N*-trimethylaminobutyraldehyde dehydrogenase (TMABADH), and γ-butyrobetaine dioxygenase (BBD). OCTN2 (organic cation/carnitine transporter novel type 2) transports carnitine into the cells. One of the major functions of carnitine is shuttling long-chain fatty acids across the mitochondrial membrane from the cytosol into the mitochondrial matrix for β-oxidation. This transport is achieved by mitochondrial carnitine–acylcarnitine cycle, which consists of three enzymes: carnitine palmitoyltransferase I (CPT I), carnitine-acylcarnitine translocase (CACT), and carnitine palmitoyltransferase II (CPT II). Carnitine inborn errors of metabolism could result from defects in carnitine biosynthesis, carnitine transport, or mitochondrial carnitine–acylcarnitine cycle. The presentation of these disorders is variable but common findings include hypoketotic hypoglycemia, cardio(myopathy), and liver disease. In this review, the metabolism and homeostasis of carnitine are discussed. Then we present details of different inborn errors of carnitine metabolism, including clinical presentation, diagnosis, and treatment options. At the end, we discuss some of the causes of secondary carnitine deficiency.

## 1. Introduction

Carnitine (l-3-hydroxy-4-*N*,*N*,*N*-trimethylaminobutyrate), is an essential water soluble molecule that has many biological functions. One of these major functions is shuttling long-chain fatty acids across the mitochondrial membrane from the cytosol into the mitochondrial matrix for β-oxidation, and hence, cellular energy production. Carnitine also modulates the acyl-CoA/CoA ratio, thereby regulating the activity of several mitochondrial enzymes [1]. The acetylated form of carnitine, acetyl-carnitine, is involved in energy storage. Carnitine conjugates with partially metabolized acyl groups and allows their excretion as carnitine esters in urine [2,3,4]. More roles of carnitine being identified include anti-inflammatory and antioxidant properties [5,6,7], and improving insulin resistance [2,8]. 

Meat, poultry, fish, and dairy products are major sources of carnitine in the diet. On the other hand, the content of carnitine in food of plant origin is low. In non-vegetarians, 75% of carnitine sources are obtained from diet, which provides 2–12 µmol of carnitine per kilogram per day (µmol/Kg/day), and the remaining 25% originates from endogenous production, which provides 1.2 µmol/Kg/day. In contrast, endogenous synthesis provides the majority (>90%) of total carnitine in strict vegetarians, in whom dietary intake provides less than 0.1 µmol/Kg/day of carnitine [9,10,11].

The vast majority (>99%) of body carnitine is situated in the intracellular compartment. Circulating carnitine represents only about 0.5% of body carnitine. Therefore, normal plasma free carnitine levels are low, ranging between 25–50 µmol/L [12]. Normal levels are variable based on age and gender. In one study of 80 healthy volunteers, mean serum-free carnitine levels in males were 41 µmol/L (range 26.4–53.4) whereas in females the mean was 39 µmol/L (range 19.2–44.5) [13]. The same study showed a positive correlation between carnitine levels (free and total) with age, although in males the correlation didn’t reach statistical significance [13]. Normal carnitine levels are maintained by balance between dietary intake, endogenous synthesis, and renal reabsorption. Based on carnitine content in food, bioavailability of dietary carnitine is about 54–86% [14]. In the kidney the majority of carnitine (90–99% of filtered load) is reabsorbed until saturation is reached. The renal threshold for carnitine excretion is around 50 μmol/L. The kidneys are very efficient in maintaining normal levels of plasma carnitine by modulating urinary carnitine excretion according to the intake from diet [14].

Carnitine inborn errors of metabolism (IEM) can be divided into disorders of carnitine biosynthesis, carnitine transport, and mitochondrial carnitine–acylcarnitine cycle. The presentation of these disorders is variable but common findings include hypoketotic hypoglycemia, cardio(myopathy), and liver disease. These manifestations occur due to energy deficiency and accumulation of fatty acids in affected organs. Secondary carnitine deficiency could develop in several IEM, as a side effect of some drugs, or due to increased excretion with tubular dysfunction or dialysis.

## 2. Carnitine Biosynthesis Disorders

**Figure 1:**
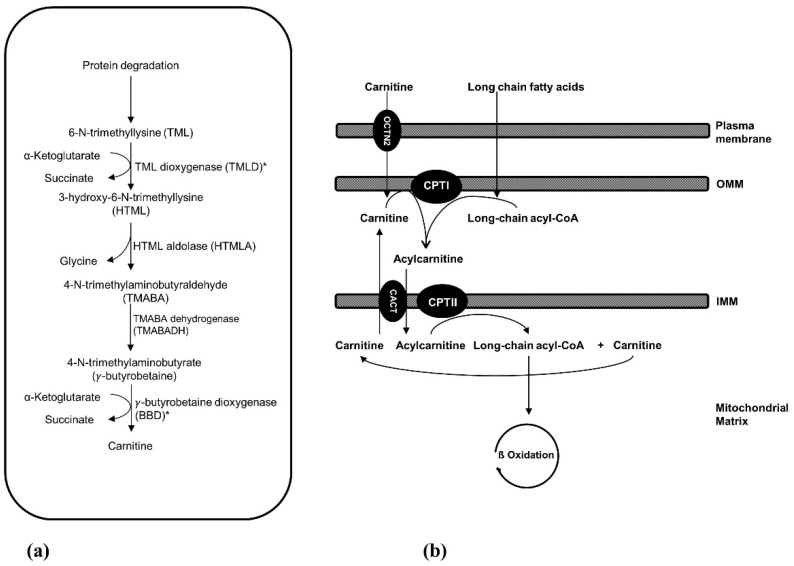
(**a**) Carnitine biosynthetic pathway. Only trimethyllysine dioxygenase (TMLD) and γ-butyrobetaine dioxygenase (BBD) deficiencies have been reported; both are denoted with asterisks. (**b**) Carnitine transport and mitochondrial carnitine–acylcarnitine cycle. IMM: inner mitochondrial membrane; OMM: outer mitochondrial membrane; CPT I: carnitine palmitoyltransferase I; CACT: carnitine-acylcarnitine translocase; CPT II: carnitine palmitoyltransferase II; OCTN2: organic cation/carnitine transporter novel type 2.

The precursors for carnitine biosynthesis are lysine and methionine which provide the carbon backbone and 4-*N*-methyl groups of carnitine, respectively. The substrate for carnitine biosynthesis is 6-*N*-trimethyllysine (TML) (Figure 1a). Certain proteins, like cytochrome c, actin, myosin, and histones, contain *N*-methylated lysine residues that are produced by post-translational *N*-methylation modification. This process is catalyzed by methyltransferases which use S-adenosylmethionine as a methyl donor. Subsequently, TML is released by lysosomal or proteasomal degradation of these proteins. The first step of carnitine biosynthesis is hydroxylation of TML by TML dioxygenase (TMLD), producing 3-hydroxy-6-*N*-trimethyllysine (HTML). The latter undergoes cleavage by HTML aldolase (HTMLA), yielding 4-*N*-trimethylaminobutyraldehyde (TMABA) and glycine. TMABA is then dehydrogenated to 4-*N*-trimethylaminobutyrate (γ-butyrobetaine) through action of TMABA dehydrogenase (TMABADH). In the fourth and last reaction of carnitine biosynthesis, γ-butyrobetaine is hydroxylated by γ-butyrobetaine dioxygenase (BBD) to produce carnitine [3,15].

Recently, TMLD and BBD deficiencies have been reported. As mentioned earlier, in individuals eating a regular diet, most of carnitine is obtained from the diet whereas carnitine biosynthesis makes a small contribution. Therefore, carnitine deficiency is not observed with carnitine biosynthesis defects, provided dietary intake is normal. The underlying pathophysiologic mechanisms in these disorders then may include abnormal levels of intermediate metabolites in the carnitine biosynthetic pathway and carnitine deficiency early in life [16]. Given the rarity of these disorders, no strictly vegetarian individuals affected with carnitine biosynthesis disorders have been yet reported. However, it is possible that carnitine levels could be low in these circumstances.

### 2.1. 6-N-Trimethyllysine Dioxygenase (TMLD) Deficiency

TMLD is a mitochondrial enzyme, encoded by *TMLHE* on the X chromosome. Celestino-Soper et al. performed array CGH (comparative genomic hybridization) in families with autism and identified *TMLHE* exon 2 deletion in a male child with autism, raising the possibility of a link between TMLD deficiency and autism [17]. Subsequently, the same group evaluated this possibility by testing a cohort of males with autism and comparing them to controls. TMLD deficiency due to exon 2 deletions was found to be common in control males (~0.28%), whereas the percentage in probands with autism was not significantly higher (~0.31%). *TMLHE* exon 2 deletions were more frequent in probands from families with more than one affected male compared to control subjects suggesting that TMLD deficiency is a risk factor for autism with low penetrance (2–4%). Cultured lymphoblastoid cell lines from males with the exon 2 deletion had low or undetectable TMLD enzyme activity. TMLD deficiency leads to a decrease in the levels of the products of the enzyme, HTML and γ-butyrobetaine, and an increase in the levels of the proximal substrate, TML [18]. In another study, next-generation sequencing in 12 families with males affected with autism identified two affected brothers who had a nonsense variant in *TMLHE*. The *TMLHE* coding sequence was then screened in 501 males with autism and 2 additional missense variants were identified [19].

A case report described a young male child with TMLD deficiency due to 2 base pairs deletion in exon 6 of *TMLHE* who regained his milestones after developmental regression and even gained new skills after initiation carnitine supplementation at 200 mg/kg/day [20]. In this child, TML levels were normal in plasma and high in urine. In contrast, γ-butyrobetaine levels were low in his plasma but within reference range in urine. The TML/γ-butyrobetaine ratio was significantly elevated in plasma and urine. The authors mentioned that this phenomenon was observed in two other individuals with *TMLHE* variants in their lab, suggesting that the TML/γ-butyrobetaine ratio may be a superior diagnostic marker for TMLD deficiency instead of TML or γ-butyrobetaine levels alone [20]. In contrary to other reports, this child reportedly had low plasma carnitine levels. Given the child was taking a normal diet, it not clear what was the reason for low carnitine levels as this cannot be explained only by TMLD deficiency. There could be a period of poor intake before the levels were measured or the child could have secondary carnitine deficiency due to an unidentified reason [16,20].

The association of TMLD deficiency with autism, and other evidence, could suggest that brain deficiency of carnitine may cause autism (in ~10%–20% of cases) and therefore early carnitine supplementation could be beneficial [21,22]. 

### 2.2. γ-Butyrobetaine Dioxygenase (BBD) Deficiency 

BBD is encoded by *BBOX1* on chromosome 11. A 42-month-old female child with BBD deficiency due to a homozygous 221 kilobases (Kb) deletion at 11p14.2 was reported. This deletion overlaps *BBOX1* and *FIBIN* encoding fibin. The girl presented with microcephaly, speech delay, poor growth and some dysmorphic features. Carnitine levels were normal. With only one case report, it is difficult to conclude causation with any of these genes [23]. In a recent study, Lee et al. showed that *BBOX 1* expression was down-regulated in a mouse model with schizophrenia that was induced by maternal immune activation. The authors also did a case control study on 284 subjects with schizophrenia and 409 healthy controls and found that *BBOX 1* polymorphisms could be associated with increased schizophrenia susceptibility in the Korean population [24].

## 3. Carnitine Transport Defect

Systemic primary carnitine deficiency, due to carnitine transport defect, is caused by biallelic variants in *SLC22A5* [25]. It has an estimated incidence of 1 in 120,000 newborns in the United states [26]. It is more common in Japan with an estimated incidence of 1 in 40,000 [27]. *SLC22A5* encodes organic cation/carnitine transporter novel type 2 (OCTN2). OCTN2 is a high affinity organic cation transporter specific for carnitine that works in Na+ dependent and Na+ independent mechanisms [28]. It is highly expressed in heart, muscle, kidneys, and other tissues. Defects in OCTN2 results in urinary carnitine wasting, low serum carnitine levels, and decreased intracellular carnitine accumulation [12].

As the major function of carnitine is shuttling long-chain fatty acids into the mitochondrial matrix for β-oxidation and energy production, carnitine deficiency results in defective fatty acid oxidation presenting as hypoglycemia due to excessive glucose consumption without regeneration via gluconeogenesis. Fat released from the adipose tissue with fasting will not be utilized and instead will accumulate. Fat accumulation in the liver, skeletal muscle, and heart can impair organs’ function and cause steatosis, cardiomyopathy, and myopathy [12].

The presentation of primary carnitine deficiency is very variable in terms of onset, severity, and involved organs. Inability to utilize fat for energy during stress or fasting can trigger acute metabolic decompensations early in life. Later presentation includes skeletal myopathy, cardiomyopathy, and arrhythmias with sudden death [12]. Still, affected individuals could remain asymptomatic [29].

Around 50% of affected individuals present before the second year of life with acute metabolic decompensations precipitated by fasting or intercurrent illnesses. During these episodes, affected individuals develop lethargy, irritability, and poor feeding. Hepatomegaly is a common sign. Hypoketotic hypoglycemia, elevated liver enzymes, and hyperammonemia, are usually evident on laboratory evaluation. Without early treatment, these episodes could progress to coma and death [16]. The remaining 50% of affected individuals present between 2–4 years of age with a more insidious presentation that includes skeletal myopathy with muscle weakness and hypotonia, elevated CK (creatinine kinase), and dilated cardiomyopathy. Cardiomyopathy in individuals with primary carnitine deficiency responds poorly to standard therapy and without accurate diagnosis and carnitine supplementation, it can be fatal [12,16,30,31]. 

Adults with primary carnitine deficiency could be asymptomatic or they could have mild symptoms with easy fatigability [16]. While cardiomyopathy is not commonly seen in affected adults, they are still at risk for cardiac arrhythmias and sudden death, even if have been asymptomatic. Affected women with primary carnitine deficiency have been occasionally diagnosed after identifying low carnitine levels in their infants on newborn screening (NBS). Half of these women were asymptomatic whereas the rest complained of fatigability. The metabolic stress associated with pregnancy could trigger symptoms in otherwise asymptomatic women [32,33,34]. 

There is a correlation between the genotype and the degree of carnitine transport in fibroblasts [35]. Rose et al. showed that carnitine transport was higher in asymptomatic women (diagnosed by abnormal NBS in their babies) compared to symptomatic individuals. They showed higher frequency of nonsense variants in those who were symptomatic [35]. However, there is usually no correlation between genotype and the occurrence of metabolic and cardiac complications as these have been observed in the presence of residual transporter activity [12]. Other factors could contribute to the phenotype in affected individuals including exposure to intercurrent stressors that trigger catabolism (e.g., fasting, infections) or worsening of carnitine deficiency by other factors (e.g., pivalic acid containing antibiotics [36]). 

Scaglia et al. showed that heterozygote carriers have partially reduced carnitine transport in fibroblasts with increased urinary losses and borderline plasma carnitine levels compared to controls [37]. Heterozygote carriers are usually asymptomatic although the carrier status could be an independent risk factor for cardiac complications. Xiaofei et al. showed that heterozygote mice developed age-associated left ventricular myocyte hypertrophy with lipid deposition and abnormal mitochondria [38]. Similarly, Takahashi et al. evaluated heterozygote mice in the presence of surgically induced hypertension. These mice showed exaggerated cardiac hypertrophy and increased mortality compared to the wild-type mice. Carnitine supplementation prevented these changes in mice [39]. These findings are further supported by studies in murine models with moderate carnitine deficiency induced by carnitine depleting agents [40,41,42]. In a cohort of Japanese individuals, heterozygote, middle-aged individuals developed mild left ventricular hypertrophy of no clear clinical significance [27]. Another study suggested that heterozygosity for S*LC22A5* variants is less likely to be an important cause of cardiomyopathy. In this study, 324 individuals with cardiomyopathy were tested for *SLC22A5* variants. The frequency of variants affecting carnitine transport was 0.61% in individuals with cardiomyopathy compared to 1.11% in the general population [43]. 

Primary carnitine deficiency is suspected in symptomatic individuals who have low free carnitine levels. Affected individuals usually have very low plasma free carnitine levels (<5 μmol/L) [12]. Alternatively, the disorder can be identified by NBS using tandem mass spectrometry [44]. The false positive rate could be high as levels of carnitine in the newborn strongly reflect maternal levels, which could be low as carnitine levels decrease during pregnancy [45]. Also, the mother could have primary or secondary carnitine deficiency (see below) which could be unmasked by abnormal screening in the newborn [46]. In a recent study from California, 1030 out of 3,608,768 newborns screened positive for low free carnitine. Out of these, only 21 cases were confirmed cases of primary carnitine deficiency and a further 27 cases were possible cases [46]. This study also showed that newborns with primary carnitine deficiency could have free carnitine levels close to the cutoff and this raises the possibly that true cases could have been missed by NBS [46]. Diagnosis of primary carnitine deficiency can be confirmed by DNA testing of the *SLC22A5* gene. If molecular testing is not conclusive, an alternative option is skin biopsy to assess carnitine transport in cultured fibroblasts [16]. 

Individuals with primary carnitine deficiency should be treated with carnitine supplementation at a dose of 100–200 mg/kg/day, usually divided three times daily [12]. The dose should be titrated according to the levels of free carnitine in plasma. Carnitine is well tolerated with only few side effects. Diarrhea and abdominal discomfort could be observed with high doses. Bacterial metabolism in the intestine can produce trimethylamine, which has a fishy odor. This side effect may respond to reducing carnitine dose; otherwise, a course of oral metronidazole and/or probiotics may be indicated [12,16]. Primary carnitine deficiency has good outcomes and favorable prognosis provided that affected individuals remain compliant with carnitine supplementation.

## 4. Mitochondrial Carnitine-Acylcarnitine Cycle Disorders

As mentioned earlier, carnitine is required for the transport of fatty acids into the mitochondrial matrix for β-oxidation. Once inside the cells, fatty acids are first activated by long-chain acyl-CoA synthetase, forming long-chain acyl-CoAs [47]. There are different long-chain acyl-CoA synthetase enzymes specific for different size fatty acids [48]. Once activated, long-chain acyl-CoAs are transported to the mitochondrial matrix through the mitochondrial carnitine–acylcarnitine cycle, which is required to overcome the permeability barrier of the inner mitochondrial membrane. This cycle consists of three steps (Figure 1b), the first of which is conversion of long-chain acyl-CoAs to their acylcarnitine equivalents through the action of carnitine palmitoyltransferase I (CPT I). This enzyme is located on the outer mitochondrial membrane. The second step is the transport of acylcarnitines into the mitochondrial matrix by carnitine-acylcarnitine translocase (CACT). In the last step, carnitine palmitoyltransferase II (CPT II) at the inner mitochondrial membrane reconverts acylcarnitines back to the acyl-CoA species and carnitine [49]. 

### 4.1. Carnitine Palmitoyltransferase I (CPT I) Deficiency

There are three isoforms of CPTI. CPT1A is called liver CPT1, but it is also expressed in the brain, kidney, and other organs. CPT1B is the muscle isoform whereas CPT1C is a brain-specific enzyme [50]. Initially, it has been thought that only pathogenic variants in *CPT1A*, coding CPTIA, are associated with disease in humans. Recently, pathogenic variants in *CPT1C,* coding CPTIC, were identified to be causative for spastic paraplegia 73 [51,52]. 

CPT1A deficiency is a rare, autosomal recessive disorder with an estimated incidence from NBS data of about 1:750,000 to 1:2,000,000 [53]. It could be more common in certain populations such as Inuit and Hutterites [54,55]. CPTIA deficiency is characterized by episodes of hepatic encephalopathy that are triggered by fasting or intercurrent illnesses. Affected individuals usually present before 2 years of age with hypoketotic hypoglycemia, hepatomegaly, and elevated liver transaminases. Without treatment, these symptoms can progress to seizures and coma [56,57,58]. Other features include renal tubular acidosis and elevated CK levels. Heart and skeletal muscle involvement is less common [56,59]. There are reports of heterozygous mothers carrying an affected fetus who presented with acute fatty liver of pregnancy (AFLP) [60].

Apart from hypoketotic hypoglycemia and raised transaminases, other suggestive laboratory findings of CPTIA deficiency include mild hyperammonemia and elevated total serum carnitine with elevated C0/C16+C18 ratio. This ratio is in fact very specific to diagnose this disease since it is elevated in both; plasma and direct blood spot (DBS) samples [61]. On the other hand, free carnitine levels are higher in DBS compared to plasma in individuals with CPTIA deficiency and therefore, if only plasma free carnitine levels are measured, the diagnosis can be missed [61,62]. Urine organic acids might show hypoketotic dicarboxylic aciduria, in particular elevated C12 dicarboxylic acid [56,63]. Diagnosis of CPTIA deficiency can be confirmed by DNA testing of the *CPT1A* gene or by documenting low CPTI enzyme activity on cultured fibroblasts if the DNA testing is not conclusive.

During acute episodes, affected individuals should receive high dextrose fluids to prevent catabolism and lipolysis. Long-term management involves frequent feeding, avoidance of fasting, and uncooked cornstarch at night. The diet should be low in fat, high in carbohydrates, and rich in medium-chain triglycerides (MCT) [56]. 

### 4.2. Carnitine-Acylcarnitine Translocase (CACT) Deficiency

Carnitine-acylcarnitine translocase (CACT) deficiency is also a rare autosomal recessive disease caused by defects in *SLC25A20.* Without the translocase, long-chain fatty acids will not be available for mitochondrial β-oxidation [64]. Most affected individuals present in the neonatal period with a severe phenotype characterized by a rapidly progressive course and a high mortality. Presenting features of CACT deficiency include hypoketotic hypoglycemia that can result in seizures and coma, respiratory distress, arrhythmia, cardiomyopathy, liver disease, and sudden death [65]. In addition to hypoketotic hypoglycemia, other laboratory abnormalities include hyperammonemia, elevated liver enzymes, and elevated CK levels. Late onset presentation with a milder phenotype has been reported but less commonly [66,67]. This phenotype presents in the form of episodes of hypoketotic hypoglycemia that are triggered by fasting or intercurrent illnesses. Milder phenotype is associated with some degree of residual enzyme activity [66]. 

CACT deficiency is diagnosed based on elevated levels of long-chain fatty acid acylcarnitine esters (especially C16 and C18:1) along with low free carnitine [65]. This biochemical pattern is indistinguishable from CPT II deficiency (see below) and therefore, either DNA testing or measuring enzyme activity in cultured fibroblasts is required to confirm the diagnosis. Urine organic acids can reveal non-specific dicarboxylic aciduria. 

Treatment involves avoidance of fasting and frequent meals. The diet should be rich in carbohydrates and low in fat. Most fat intake should come from MCT. Carnitine is commonly used, but it is controversial. There have been concerns about the possible toxicity of acylcarnitine accumulation in long chain fatty acid oxidation disorders (see Section 6).

### 4.3. Carnitine Palmitoyltransferase II (CPT II) Deficiency

CPT II deficiency is an autosomal recessive disorder caused by biallelic pathogenic variants in *CPT2*. The presentation is variable in regard to age of onset, severity, and involved organs. Three phenotypes are described including, myopathic form, lethal neonatal form, and severe infantile form [68]. 

By far, the myopathic form is the most common form of CPT II deficiency and it is a common cause for hereditary rhabdomyolysis [69]. CPT II deficiency is considered as the most common disorder of lipid metabolism affecting skeletal muscles [68]. The myopathic form of CPT II deficiency can present at any age with 70% of cases presented during childhood (0–12 years of age). In one quarter of cases, the disease presented during adolescence (13–22 years) [70,71]. The myopathic form is characterized by recurrent attacks of rhabdomyolysis that are triggered by prolonged exercise in the majority of cases. Other triggers include infections and fasting [71]. During these episodes, myalgia is the most consistent symptom, and it can be associated with muscles weakness and myoglobinuria [68,70,71], and CK levels are elevated. Episodes of rhabdomyolysis can lead to acute renal failure in 7–23% of cases [71]. The myopathic form of CPT II deficiency is more commonly observed in males, although it is an autosomal recessive disorder with expected equal gender distribution [72]. This observation could be due to ascertainment bias as males are more likely to do strenuous exercise and therefore develop myoglobinuria. Hormonal factors were also suggested to be involved in this gender bias [72,73].

The other two forms of CPT II deficiency are very rare. Affected individuals with the neonatal form present soon after birth (hours to a few days) with lethargy, respiratory distress, hypoketotic hypoglycemia, liver failure, cardiac arrhythmias, cardiomyopathy, seizures, and coma [74,75]. Affected neonates could have dysmorphic features, renal dysgenesis, neuronal migration defects, and other brain malformations [68,76]. The prognosis is poor, with death occurring within days to months [68]. 

The severe infantile form usually presents between 6–24 months of age [57]. It is characterized by hypoketotic hypoglycemia, hepatomegaly, liver failure, cardiomyopathy, and cardiac arrhythmias. Seizures and coma could develop secondary to hypoglycemia [57,68,77]. Episodes of decompensation could be triggered by fasting or intercurrent illnesses. Cardiac arrhythmias can result in sudden death [75]. In both neonatal and infantile forms, laboratory evaluation usually shows hyperammonemia, metabolic acidosis, hypoketotic hypoglycemia, and high CK levels.

CPT II deficiency is diagnosed based on elevation of C12 to C18 acylcarnitines, in particular C16 and C18:1 [68]. Free carnitine levels could be reduced. (C16+C18:1)/C2 ratio is a more sensitive marker and could improve the sensitivity of the NBS [78]. It should be noted that CPT II deficiency is better diagnosed from plasma samples compared to DBS [61]. Diagnosis can be confirmed by DNA testing or enzyme assays in fibroblast. Treatment principles are the same as discussed before for CACT deficiency. Prolonged exercise and other known triggers should be avoided [57,68]. Table 1 summarizes carnitine inborn errors of metabolism.

### 4.4. Carnitine Palmitoyltransferase (CPT) Inhibitors

While the mitochondrial carnitine–acylcarnitine cycle is essential for fatty acid oxidation and energy supply, modulation of these processes, through CPT inhibitors, could be of a potential therapeutic value in several disorders, including diabetes, cancer, and heart diseases [79]. For example, in type 2 diabetes, the underlying reduced insulin sensitivity causes increased fatty acid oxidation which by itself aggravates the hyperglycemia by reducing glucose utilization and increasing production through gluconeogenesis [80]. Selective inhibition of liver CPTI reduces gluconeogenesis and improves glucose homeostasis [81]. In the diseased heart, there is an increase in fatty acid oxidation and a decrease in glucose consumption, which in turn impair energy utilization and decrease cardiac efficiency [82]. Lionetti et al. showed that CPTI inhibition in dogs with heart failure delayed the time to end-stage failure [83]. Etomoxir, an irreversible inhibitor of CPTI, was found to prevent the development of heart failure in rats with pressure overload-induced cardiac hypertrophy [84]. Placebo-controlled trial in humans was stopped prematurely due to hepatotoxicity, although it showed trends for improved cardiac function in treated subjects [85]. Finally, through inhibition of fatty acid oxidation which fuels the tumor cells, and other mechanisms like apoptosis and modulating gene expression, CPT inhibition could be a potential target in cancer [86].

## 5. Secondary Carnitine Deficiency 

Apart from primary carnitine deficiency, which is caused by carnitine transport defect, secondary carnitine deficiency could also develop due to a variety of reasons [87]. As compared to the primary form, secondary carnitine deficiency is less severe and associated with higher carnitine levels that can be treated with small doses of carnitine [16]. Carnitine deficiency is seen in several IEM, in particular, fatty acid oxidation disorders and organic acidemias. In these disorders, carnitine deficiency develops secondary to increased urinary excretion of carnitine in the form of acylcarnitines [88]. It is uncommon to develop carnitine deficiency secondary to poor intake as carnitine biosynthesis along with renal reabsorption are effective in maintaining normal carnitine levels [89]. Still, malnutrition and malabsorption can cause carnitine deficiency. An adult individual with severe malnutrition and history of gastric bypass surgery developed hyperammonemia and was found to have very low carnitine levels. Hyperammonemia was refractory to standard therapy but normalized with carnitine supplementation [90]. Children may be more prone to dietary carnitine deficiency [89]. In particular, preterm infants are born with limited carnitine reserves as placental transfer and biosynthesis of carnitine occurs mainly during the third trimester. They also have limited ability to synthesize carnitine due to immaturity of biosynthetic enzymes [91]. Therefore, carnitine levels may need to be monitored in those neonates, especially with prolonged use of total parental nutrition [92]. However, the evidence for routine administration of carnitine in preterm infants is lacking and more studies are needed [93,94]. 

Valproic acid (VPA) is one of the well-known drugs causing secondary carnitine deficiency, which can contribute to VPA-induced toxicity [95]. VPA depletes carnitine through different mechanisms including increased urinary excretion in the form valproylcarnitine, reduction in tubular reabsorption and endogenous biosynthesis, and inhibition of the carnitine transporter [95]. Pivalic acid containing antibiotics can also cause carnitine depletion as pivalate conjugates with free carnitine, forming pivaloyl carnitine, which is excreted in the urine [36,96]. Several drugs can cause secondary carnitine deficiency though OCTN2 inhibition including omeprazole [97], zwitterionic drugs (e.g., levofloxacin) [98], and anticancer drugs (e.g., etoposide, vinblastine, actinomycin D) [99]. 

Secondary carnitine deficiency could also develop from increased urinary loss in Fanconi syndrome or with renal replacement therapy (hemodialysis and peritoneal dialysis). As mentioned earlier, the kidneys are very efficient in maintaining carnitine homeostasis. Several mechanisms contribute to carnitine depletion with chronic dialysis including efficient free carnitine removal through dialysis, decreased intake, and decreased endogenous production [100]. In individuals with end stage renal disease, free carnitine levels are higher than healthy individuals prior to initiation of dialysis. Once dialysis starts, this level declines significantly, about 30% in 4 weeks and 40% in 12 months [101]. This is associated with increased acylcarnitine and acylcarnitine/free carnitine ratio. Dialysis also results in reduction in muscle carnitine content [101]. Dialysis-related carnitine disorder (DLD) is the term used to describe carnitine deficiency in dialysis patients and can be associated with several complications including fatigue, intradialytic hypotension, anemia that is poorly responsive to erythropoietin, and cardiomyopathy [102]. Many clinical trials described the benefits of carnitine to treat these complications although definite evidence is still lacking [103]. To attain desired effects on multiple pathways, higher than physiologic levels of carnitine should be achieved in the plasma, and therefore, in the intracellular compartment. This is possible in individuals with end stage renal disease on dialysis who receive parenteral carnitine supplementation, given their underlying renal disease will impair rapid clearance of carnitine when transporter saturation reached. In this way, carnitine acts as a “conditionally essential nutrient” [104]. 

## 6. Carnitine Use in Inborn Errors of Metabolism

Carnitine supplementation is commonly used in several IEM and other disorders. It is the mainstay of treatment in primary carnitine deficiency. Carnitine is also used in other IEM that are associated with secondary carnitine deficiency [105,106], although a Cochrane review concluded that evidence for carnitine supplementation in IEM is lacking [107]. Moreover, there have been concerns about possible toxicity of acylcarnitine accumulation in long chain fatty acid oxidation disorders. In one report, two siblings with very long-chain acyl-CoA dehydrogenase (VLCAD) deficiency developed more frequent episodes of rhabdomyolysis after carnitine supplementation [108]. In a mouse model, accumulating long-chain acylcarnitines in ischemic heart mitochondria inhibited oxidative phosphorylation [109]. Long chain acylcarnitines were found to regulate the hERG (human ether-a-go-go-related gene) channel and contribute to the development of cardiac arrhythmias [110]. Based on this potential risk, the use of carnitine in long chain fatty acid oxidation disorders should be avoided during acute metabolic crises [111]. Carnitine is often used in patients with mitochondrial disorders [112]. In these disorders, plasma free carnitine tends to be lower than normal with increased esterified carnitine due to a partial impairment of β-oxidation [113]. Metabolism of carnitine through gut microbiota produces trimethylamine *N*-oxide (TMAO). Marked elevation in plasma levels of TMAO were documented in patients with mitochondrial disorders treated with oral carnitine [114]. Recent studies showed a positive correlation between elevated levels of TMAO and an increased risk for cardiovascular disease [115]. Given this potential risk and lack of evidence for the benefit of carnitine in patients with mitochondrial disorders, more studies are needed to evaluate the long-term safety and efficacy of carnitine in these patients.

## 7. Summery and Conclusions

Carnitine is involved in several biochemical pathways, either directly or indirectly. Carnitine is obtained from the diet, synthesized endogenously, and excreted in the urine. In normal situations, these processes are very efficient in maintaining normal carnitine levels. It is important to recognize inborn errors of carnitine metabolism as early treatment could prevent serious sequelae. Disorders of carnitine biosynthesis were recently identified and are still to be fully elucidated. The association of TMLD deficiency with autism is interesting and could help open venues to better understand the pathophysiology of autism, in a subset of patients. Other inborn errors of carnitine metabolism, carnitine transport defect, and mitochondrial carnitine–acylcarnitine cycle disorders, have been recognized for long time and a common theme in these disorders is energy failure with hypoketotic hypoglycemia, cardio(myopathy), and liver disease. These disorders are part of NBS programs in several areas of the world. The myopathic CPTII deficiency is a common cause for hereditary rhabdomyolysis and should be always considered with such presentations. Carnitine supplementation and the use of compounds to modulate carnitine metabolizing enzymes, like CPT inhibitors and potentiators, have been the focus of many research projects. These agents could be promising targets to treat disorders characterized by alterations in physiologic biochemical pathways, especially those concerning with energy production.

## Figures and Tables

**Table 1:** Summary of inborn errors of carnitine metabolism.

Category	Disease	Gene	Clinical Features	Diagnostic Markers	Long Term Management	References
**Carnitine biosynthesis disorders**	TMLD deficiency	*TMLHE*	• Risk factor for autism	• Low 3-hydroxy-6-*N*-trimethyllysine (HTML) and γ-butyrobetaine • High TML and TML/γ-butyrobetaine ratio	• Carnitine supplementation	[16,17,18,19,20]
	BBD deficiency	*BBOX1*	• Microcephaly, speech delay, poor growth and some dysmorphic features ^a^ • Schizophrenia susceptibility	• Not available	• Not available	[23,24]
**Carnitine transport defect**	Primary carnitine deficiency	*SLC22A5*	• Metabolic decompensations precipitated by fasting and intercurrent illnesses • Cardio(myopathy) • Easy fatigability • Asymptomatic	• Low free carnitine	• Carnitine supplementation (100–200 mg/kg/day)	[12,16,29,30,31,32,33,34,35,56]
**Mitochondrial carnitine–acylcarnitine cycle disorders**	CPT IA deficiency	*CPT1A*	• Metabolic decompensations precipitated by fasting and intercurrent illnesses • Heart and skeletal muscle involvement is less common • Acute fatty liver of pregnancy (AFLP) reported in heterozygous mothers carrying an affected fetus	• High free carnitine and C0/C16+C18 ratio	• Frequent feeding, avoidance of fasting • Low fat and high carbohydrates diet • Medium-chain triglycerides (MCT)	[55,56,57,58,59]
	CACT deficiency	*SLC25A20*	• Neonatal presentation: hypoketotic hypoglycemia, respiratory distress, arrhythmia, cardiomyopathy, liver disease, and sudden death • Milder phenotype with metabolic decompensations precipitated by fasting and intercurrent illnesses	• Elevated long chain acylcarnitines (especially C16 and C18:1) • Low free carnitine	• Frequent feeding, avoidance of fasting • Low fat and high carbohydrates diet • MCT • Carnitine supplementation ^b^	[56,64,65,66]
	CPT II deficiency	*CPT2*	• Myopathic form: recurrent attacks of rhabdomyolysis triggered by prolonged exercise, infection, fasting, and cold • Neonatal form: hypoketotic hypoglycemia, liver failure, arrhythmias, cardiomyopathy, seizures, dysmorphic features, renal and brain malformations • Infantile form: hypoketotic hypoglycemia, hepatomegaly, liver failure, cardiomyopathy, and arrhythmias	• Elevated long chain acylcarnitines (especially C16 and C18:1) • Low free carnitine	• Frequent feeding, avoidance of fasting • Low fat and high carbohydrates diet • MCT • Carnitine supplementation ^b^	[56,58,67,69,70,71,72,73,74,75]

See text for abbreviations. ^a^ The reported child has a homozygous deletion that includes a *BBOX1* gene and *FIBIN* gene. With only one case report, it is difficult to conclude causation with any of these genes (see text for details). ^b^ The use of carnitine is controversial; there have been concerns about possible toxicity of acylcarnitine accumulation in long chain fatty acid oxidations defects (see text for details).
